# Addressing Systemic Racism in Birth Doula Services to Reduce Health Inequities in the United States

**DOI:** 10.1089/heq.2021.0033

**Published:** 2022-02-02

**Authors:** Marieke S. Van Eijk, Grace A. Guenther, Paula M. Kett, Andrew D. Jopson, Bianca K. Frogner, Susan M. Skillman

**Affiliations:** Center for Health Workforce Studies, Department of Family Medicine, University of Washington, Seattle, Washington, USA.

**Keywords:** reproductive health, health care delivery, birth doulas, health disparities, health equity, health policy

## Abstract

**Purpose:** Birth doulas support pregnant people during the perinatal period. Evidence of doulas' positive impacts on pregnancy and birth outcomes, particularly among underserved populations, supports expanding access. However, health workforce-related barriers challenge the development of robust doula services in the United States. This study examined the various approaches organizations have taken to train, recruit, and employ doulas as well as their perspectives on what system-level changes are needed to redress health inequities in underserved communities and expand access to birth doula services.

**Methods:** In addition to literature and policy reviews, we conducted 16 semistructured interviews from March to August 2020 with key informants from organizations involved in training, certifying, advocating for, and employing doulas, and informants involved in state policy making. We analyzed data using qualitative analysis software to identify cross-cutting themes.

**Results:** The landscape of organizations involved in doula training and certification is diverse. In discussing their training and curriculum, interviewees from large organizations and community-based organizations (CBOs) stressed the importance of incorporating a focus on structural racism in maternal health into training curricula. CBOs specifically offered three areas of systems-level change that can help equitably grow doula services: the importance of addressing structural racism, changing the balance of power in decision making and policy making, and a cautious approach to Medicaid reimbursement.

**Conclusion:** This study provides evidence of how doula organizations move the field toward better serving the specific needs of underserved populations. It recognizes the expertise of CBOs in developing policy to expand doula services to communities in need. The information from this study highlights the complexities of facilitating consistency across doula training and certification requirements and implementing a sustainable funding mechanism while also meeting communities' unique needs.

## Introduction

Pregnant individuals of color—particularly Black and Indigenous people—experience significant disparities in birth outcomes compared with their White peers.^1^ Maternal mortality rates among Black and Indigenous populations are two to three times higher than among White pregnant persons.^2^ Such disparities are not new but have persisted and grown in recent years. Researchers have focused on individual risk factors to explain the difference in outcomes, such as insurance status, level of education, pre-existing health conditions, income level, and age.

However, adjusting for individual factors alone does not eliminate existing disparities in birth outcomes or acknowledge the detrimental effects of daily exposure to racism.^3–11^ This study examines the system-level factors, specifically organizational approaches to addressing racism, that could reduce these disparities in birth outcomes and help pregnant persons navigate the health system to get equitable perinatal health care.

Researchers have associated the stress resulting from daily exposure to racism with higher rates of preterm births and low birthweight,^10,12,13^ depression and post-traumatic stress disorder,^14^ and lower odds of breastfeeding.^9^ Structural racism—macrolevel social constructs that produce and maintain inequities within populations of color in the United States—is particularly pervasive in health care.^7,12^

Underserved communities of color have reported greater incidences of delayed care, gaps in communication, brief encounters with providers, and extended wait times for appointments when compared with White populations.^3,15,16^ Pregnant people of color encounter racist structures inside and outside the health care system, including neighborhood segregation, systematic disinvestment in predominantly Black neighborhoods, and lack of access to high-quality health services.^5^

Community-based birth doula services can positively impact birth outcomes, especially those of Black and other underserved populations. Birth doulas provide physical, emotional, and informational assistance during the perinatal period, which has been reported to decrease maternal stress, lower rates of cesarean sections, and increase satisfaction with the birth experience.^17,18^ Community-based doulas offer additional support to address the experiences and effects of structural racism for pregnant individuals.^19–21^ Having access to doulas who understand and have experienced the effects of systemic racism increases a pregnant person's trust and engagement in care and strengthens their agency in decision-making processes.^7,16, 22,23^

As doulas have contributed to improved birth outcomes, researchers and policy makers have pressed to expand access to their services. However, few studies have examined the challenges doula organizations in the United States face in meeting that demand and their role in facilitating doula training and employment, specifically for doulas working with underserved populations. Our study aimed to fill this gap. Doula organizations can play a crucial part in addressing the lack of diversity in training and employment of doulas.

Major organizations have historically grounded their training in the experiences of White upper-class women, rarely including topics specific to the needs of underserved communities.^22–24^ By incorporating training topics such as advocacy, structural racism, and discrimination in care delivery, organizations can address the specific barriers underserved populations face.^7,25,26^ Our study examined the various approaches doula organizations have taken, and their perspectives on what system-level changes are needed, to redress health inequities in underserved communities, and expand access to birth doula services.

## Methods

For this descriptive study, we reviewed published and gray literature on birth doulas in the United States. Through web searches and reviewing key reports, we identified >200 organizations across the United States involved in doula care. These were organizations that train or certify birth doulas; employ doulas across a variety of work environments; and are involved in research, policy, or advocacy for doulas. Our list included training and certifying organizations, hospitals, and community-based doula organizations in underserved communities. We also sought input from experts familiar with key policy issues related to doula scope of practice and financial reimbursement.

We conducted 16 (of 37 invited for an interview) semistructured 60-minute interviews with key informants representing a variety of perspectives and experiences from March to August 2020. We interviewed executive directors, program directors, or other high-level individuals within the organizations of interest. We asked how organizations describe the role and scope of doula work, how they recruit doulas, the core competencies they cover in training, the skills and competencies they require for doulas working in underserved communities, and the barriers and challenges facing organizations and the doula workforce. The study was reviewed and approved by the University of Washington Institutional Review Board.

### Data analysis

**FIG. 1.**
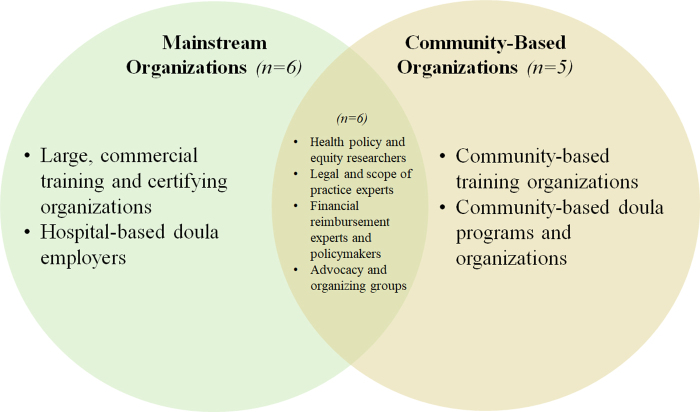
Conceptual framework for interviewee classification. The number of interviewees in each category exceeds the total number of interviews conducted as some individuals spanned multiple categories. For mainstream organizations, there was very little or no overlap between organizations that train doulas and organizations that employ doulas to work in hospital-based settings. For community-based organizations, we often found overlap between organizations that train doulas and those that employ doulas to work in community-based settings.

We used qualitative software to analyze interview transcripts using thematic analysis. Research team members independently read transcripts to identify potential themes. Informed by research and interview questions, codes were reviewed and finalized together, producing major themes and subthemes. Based on our data collection and analysis, organizations often fell into two prevailing conceptual classifications (Fig. 1). One classification included organizations that view doulas as part of the mainstream U.S. health care system and are involved in training, certifying, and employing doulas within hospital-based settings. Six such “mainstream” organizations were interviewed, including four commercial training organizations and two hospital-based doula employers.

The other classification included organizations involved in training, certifying, and/or employing doulas who are rooted in underrepresented communities to work outside of the hospital-based system, which we refer to as “community-based organizations” (CBOs). We interviewed five CBOs: two that provided doula training, two that trained and employed doulas, and one that employed doulas (but did not provide training), all specific to work in community settings. We also interviewed six experts in the legal, policy, and financial reimbursement aspects of the profession, which we classified as working at the intersection of mainstream and community-based organizations.

## Results

Major themes that participants described demonstrate (1) the diversity of approaches and perspectives with respect to training, recruitment, and funding, as well as (2) three areas for systemic change. Results for the first theme contextualize the three priority areas that can help equitably grow doula services.

### A diverse doula landscape of training, recruitment, and funding

Mainstream organizations are those that train, certify, and/or employ doulas to work primarily in hospital-based environments. Two of the six mainstream training organizations interviewed also provided an optional certification for doulas to pursue and did not employ individual doulas. The two mainstream employer organizations in our sample tended to exclusively hire doulas that came from mainstream training organizations to work primarily in hospital settings, with a preference for doulas who were certified.

With respect to the CBOs in our sample, the four CBOs that both trained and employed doulas to work in their communities offered a full training for new doulas and/or additional instruction to doulas who had already completed training with a mainstream organization. CBO doula employers placed less of an emphasis on certification as a hiring qualification. In sum, the organizations' different stances toward training, certification, and recruitment illustrate the diversity of the doula landscape and featured prominently in the conversations around systemic change illustrated hereunder.

#### Training

organizations involved in involved in developing the doula workforce is diverse. Mainstream training organizations offered shorter courses to equip doulas to work independently in primarily, but not exclusively, hospital-based settings. They frequently incorporated entrepreneurship and business skills into their curricula. These mainstream organizations also offered optional certification for doulas to pursue.^a^ CBOs' longer programs often focused on racial and reproductive justice and health inequities.

Across organization types, interviewees stressed the importance of incorporating content that addressed the impacts of structural racism on pregnancy and birth outcomes. Some mainstream training organizations had yet to make any changes, whereas others worked to center their training less on the birth experiences of White middle-class communities and conventional clinical environments by bringing in trainers of color, diversifying leadership, and building the organization's internal awareness of racism and other forms of discrimination. “The work is continuing” one interviewee emphasized, recognizing that changing an organization from within and including a focus on health equity and racism take time and require continuing education for everyone involved.

Community-based training organizations, in contrast, have historically had a strong focus on inclusivity and addressing the White centeredness of U.S. hospital care. Their trainings focused on the effects of discrimination on birth outcomes and were tailored to the needs of specific communities. For instance, one CBO started a training program for doulas who wanted to work with Native American communities and address the systemic challenges they faced, because doulas did not learn about communities' poverty and substance misuse in mainstream training programs. A few interviewees stressed the need to increase utilization of CBOs in training doulas to work in underserved communities, rather than overhaul mainstream trainings.

#### Recruitment

Interviews revealed that the diversity of training and recruitment is, next to an absence of standardized certification requirements, the result of different visions of a doula's role in the United States health care system. Hospital-based employer organizations aimed to recruit doulas who matched the racial/ethnic backgrounds of the clients they served and who could work collaboratively within a hospital's birth team.

Interviewees from CBOs employing doulas focused on recruiting doulas who were committed to serving, and had lived experiences in, underserved communities, and a mindset toward racial and social justice and health equity. As one interviewee from a CBO stated:

The prerequisite is your mindset and commitment to the work and to your ability and capacity to learn. There's also the prerequisite of being Black. We are an unapologetically Black organization dedicated to addressing Black families and the health in our communities.

#### Funding

Both mainstream organizations and CBOs struggle to fund their programs. Few had access to continuous sustainable funding sources. Specifically, mainstream employers interviewed used a variety of funding mechanisms. One relied on volunteer doulas after a grant ended. Another subsidized services for low-income clients with funds from private-pay clients limiting the number of lower income clients they could serve.

Most CBOs in our sample relied on grant funding. One organization contracted with accountable care organizations under its state Medicaid plans. Interviewees stressed that “living and dying by grant funding cycles” was unsustainable for their organizations and the communities they served. They struggled to write and submit grant applications without impacting other aspects of their work. Interviewees reported that the lack of funding forced them to balance long-term accountability to their communities with short-term grant funding that limited their ability to see a tangible impact.

### Three areas of systems-level change

Mainstream organizations and CBOs called for training that meets the needs of diverse populations. In addition, interviewees from CBOs offered three areas of systems-level change to help grow doula services while equitably serving communities: acknowledge racism as causing pregnancy-related health inequities, change the balance of power across organizations, and approach Medicaid reimbursement expansion with caution.

#### Acknowledge racism as the cause of pregnancy-related health inequities

Interviewees from CBOs pointed out that centering on doula service expansion as the sole answer to reducing pregnancy-related health inequities was unrealistic when the U.S. health system's racist structures were left unaddressed. One interviewee said:

We have to address institutional and systemic racism… [or] we are going to continue to get the birth outcomes we are getting. Because the risk factor is not your race, it is racism. It is not your ethnicity; it is the racism. And I think not enough people are paying attention to that.

Many interviewees gave examples of the harm they witnessed among their non-White clients in hospitals.

Having been a birth doula for three years, I have witnessed incredibly disrespectful care for clients who were not White. When you talk about birth trauma and disrespectful or inhumane care, most doulas can describe in detail what they have witnessed their clients go through…We need to train and employ doulas to represent the populations in our country.

Interviewees argued that placing doulas squarely within this system, without addressing its systemic racism, would not offer the kind of care underserved (particularly Black and Indigenous) communities need.

#### Change the balance of power across organizations

CBO interviewees and experts stressed the need to shift the balance toward equitable representation of doulas and community-based models in decision- and policy-making processes. They highlighted the importance of a participatory process in developing state and national policies regarding funding, scope of practice, and training. One interviewee argued:

It's really locating the locus of control in [CBOs] and ensuring that any legislative mandates and any reimbursement monies are adequate and do not come with red tape that takes away the integrity of the model. As an equity transformation measure, there must be a level of trust that lets communities determine what is best for them and… empower the pathways through redistribution of resources.

CBOs discussed their struggle with receiving adequate policy and financial recognition of their community-based models and needs. They noted that reliance on external and infrequent funding invites additional parties to the table, each with their own agenda and goals around how to run doula programs and expand doula services. One interviewee stressed that CBOs risk serving the agenda of others rather than their own communities:

[Examples of red tape that community-based organizations experience] are an inadequate amount of resources being allocated… [a] mandate or scope that does not match the resources… [or] having community-based organization[s] providing a list of things to make a solid program and then funders or legislators picking and choosing only a couple and leaving others out. What the CBO was presenting was a package, not individual a la carte pieces.

Interviewees from CBOs stated that a policy approach is needed that shifts power toward CBOs and acknowledges their expertise in providing tailor-made community-based doula services.

If we can reframe our way of thinking about these systems [of doula service delivery] to be people-oriented and holistic in nature, doulas become essential to that movement.

CBOs also recognized the need for the field to come together to successfully expand doula services, stating that current divisions have negatively impacted the profession's national recognition.

We are very segmented; we each do things our own way… if we continue to go that route, we will not receive recognition. We will not receive validation. We will not be able to guide that conversation.

Interviewees noted examples of doulas gaining recognition through representation on state-level leadership and advisory councils. The field can only move forward successfully when doulas' voices are part of the decision-making process, interviewees stated.

[Rather than having outsiders standardize rules] across the nation, what would it look like to take the communities, childbearing professionals and doulas, and ask them what they think the scope of practice should be at a state level.

#### Cautious approach to Medicaid reimbursement

Across organization types, interviewees mentioned recent policy changes to expand Medicaid to cover doula services. Interviewees from hospital-based organizations tended to advocate for Medicaid reimbursement to sustain funding and help lower income communities access doula services.

If insurance providers and Medicaid can't find a way to subsidize that and get into the populations that need them, it's always going to be a service for wealthy people who can afford the cost (…) I just don't know how doulas can make a living and serve the populations that they want to without medical insurance paying for it.

Interviewees from CBOs acknowledged that Medicaid reimbursement could improve clients' access and doulas' financial sustainability. However, they were concerned that Medicaid reimbursement would create a scope of practice that would not align with the needs of their doulas or communities and would push the expansion of doula services deeper into the hospital hierarchy without addressing the systemic racism embedded in it. They worried that the difficulty of securing reimbursement through Medicaid would discourage doulas from serving these populations.

Once public money is going into something… it perpetuates systemic racism and the people that need to be doulas and working in the communities experiencing the highest levels of maternal mortality get locked out… a fear among those who do community-based work is that when the system steps in and creates structures that the system already has structured racism and classism.

## Discussion

The training and education landscape for doula services is changing rapidly as organizations recognize the need to address health inequities in birthing outcomes and the dominance of White-centered birthing experiences in doula care. This study provides evidence of the work that mainstream organizations and CBOs undertake to move the field toward better serving underserved communities. Mainstream training organizations are working to diversify their services and change curriculum.

CBOs have historically operated with a social justice and antiracist lens, which researchers have identified as important in addressing the effects of structural racism on communities of color.^5,29^ This lens is often missing from the trainings of mainstream organizations. Thus, CBOs are well positioned, when adequately supported, to train doulas who are rooted in and connected to the communities they serve, increase capacity for equitable services for all, and potentially bring increased attention to discriminatory or harmful care that pregnant people of color experience.^30^

Interviewees stated that Medicaid funding can increase access to doula services, but it is not the panacea to reducing barriers around affordability. Several states have implemented or are investigating Medicaid reimbursement policies, although with mixed results.^31^ Medicaid reimbursement can provide organizations with a steadier income and improve the affordability of doula care. Still, CBOs worry that Medicaid expansion could emphasize hospitals as being the main location for labor and birth, moving attention, expertise, and funding away from perinatal services in local communities.

They stressed that doulas may be reluctant to take on Medicaid clients because reimbursements are low, and paperwork is complicated. Their worries are shared by researchers who also cite, among several additional barriers, low Medicaid reimbursement rates, as well as conflicting certification requirements, which make it hard for doulas to become enrolled providers with managed care organizations.^26^ Interviewees felt that more community input and participation in policy making were needed, especially regarding future potential Medicaid reimbursements.

Rather than describe explicit funding solutions, CBOs proposed systems changes that are needed to ensure such solutions are developed with equitable doula representation and community involvement. In moving forward, CBOs stressed the importance of naming and addressing the effect of racism on birth outcomes and shifting the balance of power to place CBOs at the center of the policy-making process. In explicitly pointing to racism, CBOs re-emphasize the understanding that focusing on individual risk factors without unpacking the underlying context is insufficient to address inequities in birth outcomes.

CBOs' discussion of the need for a power shift aligns with researchers who have argued that l to engage in effective health equity work, organizations must exemplify equity internally by evaluating and changing policies related to decision making, resource allocation, and governance.^32–34^ Such organizations also understand the importance of continued engagement, re-education, and cross-sectoral collaboration to sustain structural and policy change.^34,35^ To transform the way pregnant people are cared for and treated, foundational work is needed that addresses systemic racism, health inequities, and community involvement to create sustainable improvements in pregnancy-related inequities.

### Limitations

This study took an organizational-level focus and thus we did not analyze the work of individual doulas. Individual doulas would be able to provide a deeper additional perspective with respect to serving underserved communities and doula training, recruitment, and employment. In addition, specific regions of the United States were underrepresented in this study, not by design, but due to challenges in recruitment during the coronavirus disease 2019 pandemic. Moreover, our aim was not to offer precise representation, but to explore the barriers and challenges organizations face when seeking to provide and expand doula services in the United States.

## Conclusions

The findings of our study highlight the (1) differing approaches to doula training, certification, and recruitment, (2) current funding constraints, and (3) systems-level changes through which to implement a sustainable funding and policy environment for CBOs to engage with the unique needs of underserved communities. As work continues in developing policy focused on expanding doula services, this study aims to increase the field's recognition of the role and expertise of CBOs, placing them at the center of such work. Future research is needed to understand how to best scale up training so that all doulas have access, while recognizing the specific needs of each individual community.

In addition, research is needed to understand the barriers and challenges that individual doulas face in navigating the U.S. health care system. Our current study addresses this need by investigating the motivations of doulas who work with underserved populations, the barriers they encounter, their strategies to mitigate the effects of those barriers, and their desired relationship with the larger U.S. health system.

## Abbreviation Used

CBOs: community-based organizations

## Notes

^a^
Certification often requires additional training, including the completion of reflection essays, physiology examinations, reflections on attended births; evaluations from medical personnel or other birth attendants for those births; additional fees for training; and, service hours or job shadowing.^27,28^ Certification can take from 3 months to a year and cost thousands of dollars if a doula receives a mentor or shadows a professional doula on the job. The time doulas invest for certification is often unpaid. In some cases, a training/certification organization oversees the certification, offering mentorship and support. In others, doulas are on their own. Organizations across the two types offered certification as an option for doulas who had completed their respective trainings, but mainstream organizations placed greater importance on certification than do community-based organizations.
